# Back-Propagation Neural Network Optimized by K-Fold Cross-Validation for Prediction of Torsional Strength of Reinforced Concrete Beam

**DOI:** 10.3390/ma15041477

**Published:** 2022-02-16

**Authors:** Zhaoqiu Lyu, Yang Yu, Bijan Samali, Maria Rashidi, Masoud Mohammadi, Thuc N. Nguyen, Andy Nguyen

**Affiliations:** 1 School of Civil and Environmental Engineering, University of Technology Sydney, Ultimo, NSW 2007, Australia; zhaoqiu.lyu@gmail.com (Z.L.); thuc.nguyen@uts.edu.au (T.N.N.); 2 Centre for Infrastructure Engineering, Western Sydney University, Penrith, NSW 2751, Australia; m.mohammadi@westernsydney.edu.au; 3 School of Engineering, University of Southern Queensland, Springfield Central, QLD 4300, Australia; andy.nguyen@usq.edu.au

**Keywords:** back-propagation neural network, genetic algorithm, k-fold cross-validation, torsional behavior, reinforced concrete beam

## Abstract

Due to the limitation of sample size in predicting the torsional strength of Reinforced Concrete (RC) beams, this paper aims to discuss the feasibility of employing a novel machine learning approach with K-fold cross-validation in a small sample range, which combines the advantages of a Genetic Algorithm (GA) and a Neural Network (NN) to predict the torsional strength of RC beams. This research study not only utilizes the application of a Back Propagation (BP) neural network and the Gene Algorithm-Back Propagation (GA-BP) neural network in the prediction of the torsional strength of the RC beam, but it also investigates neural network parameter optimization, including connection weights and thresholds, using K-fold cross-validation. The root mean square error (RMSE), mean absolute error (MAE), mean square error (MSE), mean absolute percentage error (MAPE) and correlation coefficient (R^2^) are among the evaluation metrics used to assess the performance of the trained model. To elaborate on the superiority of the proposed network models in predicting the torsional strength of RC beams, a parametric study is conducted by comparing the proposed model to three commonly used empirical formulae from existing design codes. The comparative findings of this research study demonstrate that the performance of the BP neural network is highly similar to that of design codes; however, its accuracy is inadequate. After improving the weights and thresholds by k-fold cross-validation and GA, the prediction of the BP neural network shows higher consistency with the actual measured values. The outcome of this study can be used as a theoretical reference for the optimal design of RC beams in practical applications.

## 1. Introduction

Reinforced Concrete (RC) is a complex construction material due to the complexity of its properties and high maintenance conditions. In the past few years, a huge number of studies have been conducted on the RC beams for shear and flexural capacities, but fewer are reported about the torsional strength. Several empirical/analytical formulae from structural design codes (e.g., ACI-318-14, TBC-500-2000, BS-8110, JSCE-04, CSA-14, etc.) are available for calculations of the torsional strength of RC beams. In these models, at least 10 design parameters related to members’ dimensions, reinforcement arrangement and material properties are normally required to arrive at a more accurate calculation, including the section size of the RC beam as well as longitudinal and transverse reinforcements. The design codes on the prediction of torsional strength of RC beams provide various calculation formulations in different regions. The American design code (ACI-318-14) [1] ignores the contribution of concrete to torsional strength and only considers the role of transverse and longitudinal reinforcement. The Canadian standard [2] is similar to ACI-318-14 [1]. In addition to this, the Turkish standard [3] and British standards [4] are commonly used, where the calculation of the torsion angle has been simplified. In the Japanese standard [5], the maximum torsional strength of RC members is assessed based on the ratio of torsional reinforcement. Based on these codes, the strength of RC structures has a reference value. It is important to note that the limits of the codes are increased in different cases. Therefore, the application of the codes needs to be determined on a situational basis. Additionally, based on a large number of research studies for RC structures, the form of stress combinations, initial crack angles, dislodgement of concrete, aggregate damage, etc., are continuously incorporated into the calculations and optimized to obtain better results [6,7,8,9]. This literature provides more accurate predictions but also increases the complexity of calculating the torsional strength of RC beams.

In recent years, machine learning (ML) technology has been widely developed and applied to various scenarios of force analysis of RC beams. Abdulkadir et al. [10] used genetic programming to simulate the RC beam torsional strength and proposed an empirical formulation. Additionally, ML methods such as decision trees, random forests and fuzzy logic were used to simulate the compressive strength and slump of concrete with high accuracy [11]. Ling et al. [12] employed k-fold cross-validation to optimize a support vector machine (SVM) to reduce the average relative error and improve the prediction accuracy in predicting the degradation of concrete strength. In addition, neural network-based models have gained more attention due to their autonomous learning capability and their ability to ignore parameter classes. Tanarslan and Kumanlioglu [13] collected the parameters of 84 RC beams and improved the accuracy of the ANN model, which achieved excellent prediction accuracy in comparison with national guidelines. In addition, Hosein et al. [14] and Yang et al. [15] trained a neural network model to predict the shear strength of RC beams and showed high accuracy. Amani and Moeini [16] selected six significant parameters of RC beams as input of the BP neural network and the adaptive neuro-fuzzy inference system (ANFIS) to predict the shear strength of RC beams. The prediction accuracy of ANN and ANFIS was found to be more accurate than the ACI code. In the case of RC beams under torsion, Arslan [17] applied an artificial neural network to predict the ultimate torsional strength of beams and compared the results with design code calculations. The results showed that ANN outperformed design code in predicting the torsional strength and confirmed the potential feasibility of ANN in predicting the torsional strength of RC beams. On the other hand, the optimization of neural networks has been studied by many researchers, and different types of optimization algorithms have been derived. Among ANNs, the back-propagation neural network has also been applied in engineering applications. Lv et al. combined a BP neural network and the Grey model to predict the settlement of foundation [18]. Wu et al. [19] mentioned the common problems of the BP neural network, i.e., the inaccuracy of initial weights and thresholds, which affect the accuracy of the algorithm prediction, and used GA to optimize the BP neural network to improve the accuracy in the problem of energy consumption of copper electrowinning by 14.25%. Based on this, Liu et al. [20] used the Grey Verhulst model to improve the GA-BP neural network model and stated an accurate model in settlement prediction. Furthermore, Cevik et al. [21] used genetic programming for modelling torsional strength, and Ilkhani et al. [22] proposed a novel approach to predict the torsional strength of RC beams. In addition, Arslan [23] compared the prediction of the torsional strength of RC beams between ANNs and different design codes for the research feasibility of ANN. In the ML modelling approaches, fuzzy logic, random forests and support vector machines have been reported in predicting concrete mechanical properties such as compressive strength and elastic modulus that are largely consistent with the simulation results of neural networks [11,24,25]. However, these methods, except neural networks, usually require a significant computational effort in finding an optimal solution to a complex problem. Therefore, neural networks have been used for complex nonlinear problems such as the shear strength and torsional strength of RC beams in civil aspects by researchers.

Although the optimized neural networks employed in previous studies generated positive results in the engineering field, there are few applications of neural networks in the prediction of torsional behavior of RC beams, particularly in terms of derived neural networks such as BP neural networks, GA-BP neural networks, convolutional neural networks, etc. Moreover, the BP neural network has limitations regarding optimizing weights and thresholds when the testing and validating sample datasets are insufficient. Therefore, in this paper, the k-fold cross-validation method is used to select the best model and collect its thresholds and weights as the initial values, which can significantly improve the error correction of the BP neural network model. Then, GA is utilized to optimize the weights and thresholds to improve the accuracy of the model. In addition, this paper also discusses the variations in the prediction accuracy of the BP neural network and the GA-BP neural network optimized by k-fold cross-validation for the torsional strength of RC beams. Furthermore, five statistical evaluation metrics (RMSE, MAE, MSE, MAPE and $R^{2}$) are employed to appraise the prediction accuracy of the developed models. It is found that the prediction accuracy of the BP neural network improves when optimized thresholds and weights are extracted and entered using the k-fold validation method. However, it is discovered that this approach has less impact on the GA-BP neural network model. In addition to this, the design codes from different sources such as ACI-318-2014 [1], TBC-500-2000 [3] and BS8110 [4] are used to predict the results and compare them with the results predicted by the model of the BP neural network.

## 2. Data Collection and Analysis

**Figure 1:**
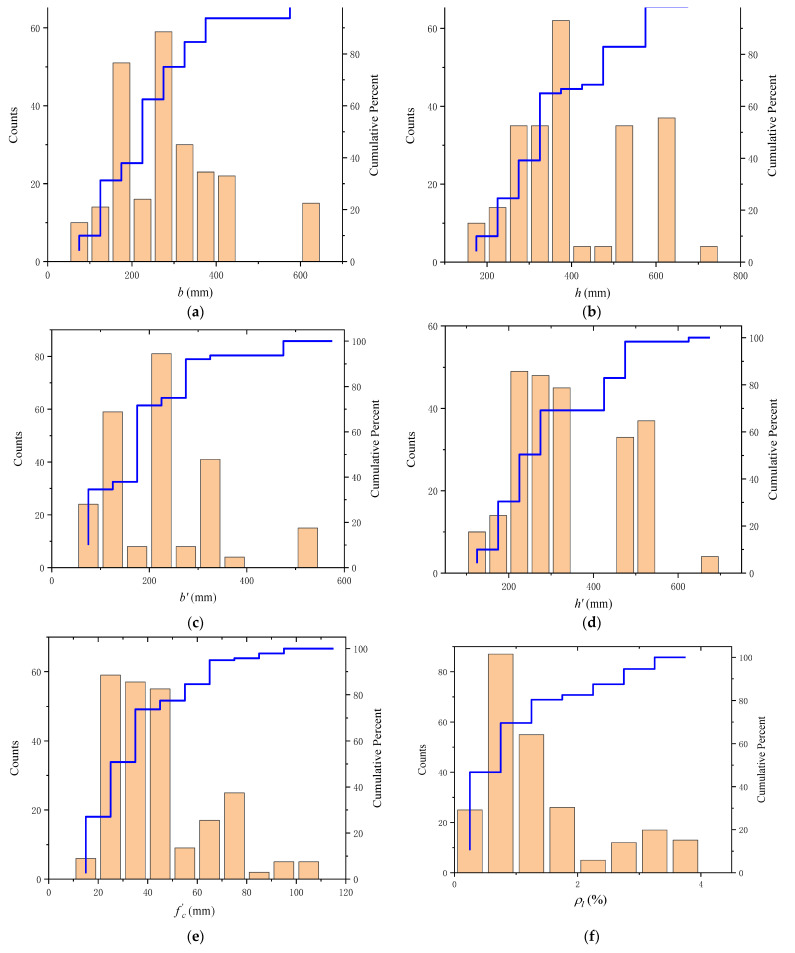

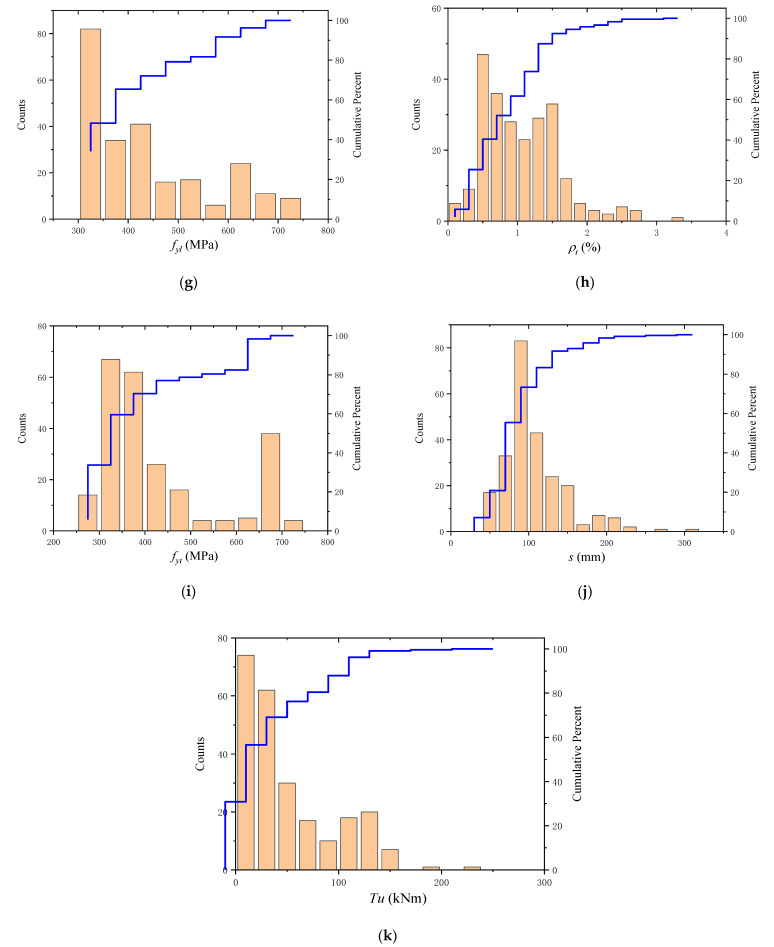
Historical distributions of parameters (**a**) RC beam section’s width (b); (**b**) RC beam section’s depth (h); (**c**) Closed stirrup width ($b^{\prime }$); (**d**) Closed stirrup depth ($h^{\prime }$); (**e**) Compressive strength ($f_{c}{}^{\prime }$); (**f**) Longitudinal reinforcement ratio ($\rho_{l}$); (**g**) Yield strength of the longitudinal reinforcement ($f_{yl}$); (**h**) Transverse reinforcement ratio ($\rho_{t}$); (**i**) Yield strength of transverse reinforcement ($f_{yt}$); (**j**) Closed stirrup spacing (*s*); (**k**) Torsional strength ($T_{n}$).

A high-accuracy BP neural network requires a large amount of data to train the model and test the model with new data samples. Since the experimental data on the torsional strength of the RC beam are limited, it is necessary to make adequate use of the available data for each parameter in order to improve the accuracy of the model. Liu [18] mentioned that BP neural network models need to consider the relative parameters of the actual problem. Additionally, according to [13,26,27], in a neural network model for predicting the strength of RC beams, a few input neurons can make the network fitting process more complex and difficult, or even fail. Therefore, in this paper, 11 different parameters of RC beams were selected, which include the RC beam section (the width (b), depth (h)), closed stirrup (width ($b^{\prime }$), depth ($h^{\prime }$), spacing (s)) compressive strength ($f_{c}{}^{\prime }$), yield strength of the longitudinal reinforcement ($f_{yl}$), longitudinal reinforcement ratio ($\rho_{l}$), yield strength of transverse reinforcement ($f_{yt}$), transverse reinforcement ratio ($\rho_{t}$) and torsional strength ($T_{u}$). The detailed information of the dataset used in this study is shown in Table 1 and Figure 1, respectively, which are collected from references [2,3,17,27,28,29,30,31].

**Figure 2:**
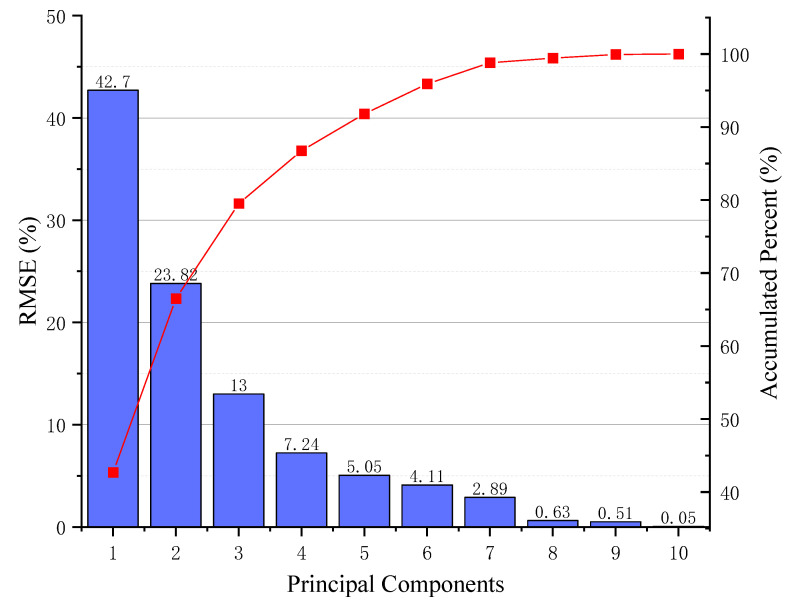
Significance of principal components.

In general, the inputs of the neural network should have small correlations between themselves. A number of the strongly correlated coefficients can lead to worse predictions of the BP neural network model, if all 10 variables are employed as inputs in this research. This is a result of the possible strong correlation of variables. Campbell and Atchley [32] suggested using the mathematical tool principal component analysis (PCA) to reduce the number of correlated variables and transform the correlated variables in the dataset to uncorrelated variables. Furthermore, PCA revealed the importance ranking of the newly generated 10 principal components (PCs). The PCA results are shown in Table 2. The first seven PCs are sufficient to represent approximately 99% of the information in the original dataset. Therefore, these seven PCs were selected as the inputs of the BP neural network. Although the number of model inputs is reduced, the quality of the data can be improved due to non-correlation, as shown in Figure 2.

## 3. Methodology

### 3.1. Design Code

Due to the building standard differences in various regions, three widely used design codes are selected as comparison candidates. The details of these codes are shown in Table 3. In addition, according to the applicable conditions of the design codes, some parameters are limited, and calculation results may generate deviations.

### 3.2. K-Fold Cross-Validation

**Figure 3:**
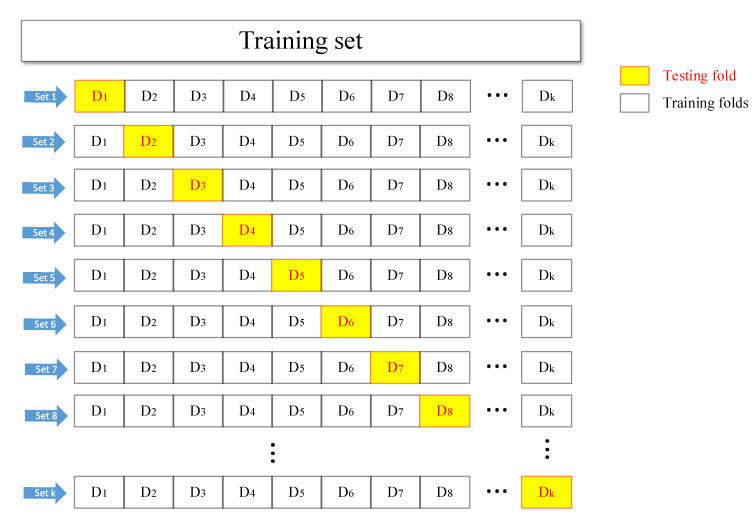
The main process of K-fold cross-validation.

The flow chart in Figure 3 shows that the k-fold cross-validation starts by randomly breaking up the data into K groups, after which, for each group, the following operations are performed:

- Select one of the training folds as the testing dataset.
- The remaining K−1 groups are used as the training set.
- Use the selected training dataset to train the model and evaluate it with the testing dataset.

In a small sample dataset of this work, k is usually set as 10, which is an empirical value obtained through extensive experimental trials. Directly utilizing the neural network simulation results in low bias and modest variance of the outcome. Therefore, in this simulation, the comprehensive datasets were randomly divided, with the first 170 sets selected as the training set and the last 70 sets as the testing set. Then, 170 samples were divided into 10 training folds. In addition, a different testing fold from D1 to D10 was selected each time as the validation set. Afterward, these 10 sets of data were inputted into the BP neural network model sequentially. The inaccuracy of the model evaluation caused by the accidental division of the sample datasets can be excluded via 10-time cross-validation.

### 3.3. BP Neural Network and Genetic Algorithm

Based on the advantages of the BP neural network, such as the nonlinear mapping capability, self-learning and self-adaptive capability, generalization capability and fault tolerance, this paper discusses the applicability of the BP neural network in predicting the torsional strength of RC beams. The forward and backward computation refers to [26]. 

The activity level for neuron *j* in layer *l* is

(1) $$v_{j}^{\left(l\right)}\left(n\right)=\sum_{i=0}^{p}w_{ji}^{\left(l\right)}\left(n\right)y_{i}^{l-1}\left(n\right),$$

The logic sigmoid function for threshold is

(2) $$y_{j}^{\left(l\right)}\left(n\right)=(1+\exp{\left(-v_{j}^{\left(l\right)}\left(n\right)\right)}^{-1},$$

The weight of the neural network is

(3) $$w_{ji}^{\left(l\right)}\left(n+1\right)=w_{ji}^{\left(l\right)}\left(x\right)+\alpha \left[w_{ji}^{\left(l\right)}\left(n-1\right)\right]+\eta \delta_{j}^{l}\left(n\right)\cdot y_{j}^{\left(l-1\right)}\left(n\right),$$

where δ in the output layer and hidden layer are, respectively,

(4) $$\delta_{j}^{\left(l\right)}\left(n\right)=e_{j}^{\left(l\right)}\left(n\right)\cdot o_{j}\left(n\right)\left[1-o_{j}\left(n\right)\right],$$

(5) $$\delta_{j}^{\left(l\right)}\left(n\right)=y_{j}^{\left(l\right)}\left(n\right)\left[1-y_{j}^{\left(l\right)}\left(n\right)\right]\sum_{k}\delta_{k}^{\left(l+1\right)}\left(n\right)w_{kj}^{\left(l+1\right)}\left(n\right),$$

and the experience of α is chosen between 0 and 1 and the learning rate η=0.5, which is suggested by [33,34].

**Figure 4:**
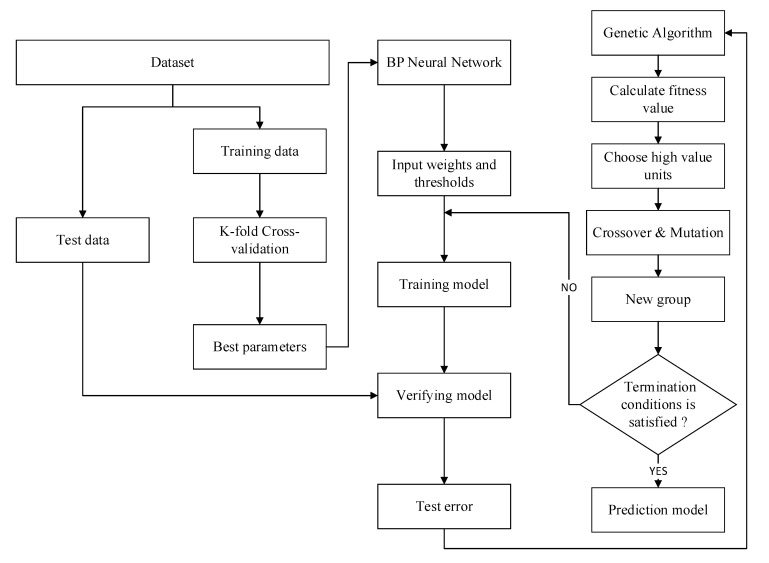
Flow chart of BP neural network optimized by K-fold cross-validation.

In a BP neural network, the neural network has a nonlinear mapping capability, which is suitable for solving problems with complex mechanisms, so the neural network can predict the nonlinear function output. It can obtain random weights and thresholds from the divided samples and start training the model. Using the BP algorithm, the partial derivative (gradient) of the loss function with respect to the weights and biases of each layer is found based on the loss function [33]. Then, this value is used to update the initial weights and bias terms until the loss function is either minimized or the set number of iterations is completed. In addition, this value is also used to calculate the best parameters for the neural network. The next part is the genetic algorithm section, which calculates adaptation values, crossover, variation and other steps to select the best group until it is close to the optimal solution [35,36,37]. In general, the GA uses a binary code and divides the program into four parts: Input and hidden layer link weights, hidden layer weights, hidden and output layer weights and output layer weights. Each weight and threshold are encoded in M-bit binary and then the optimized weights and thresholds are fed into the BP neural network. Figure 4 demonstrates the flowchart of BP neural network optimized by K-fold cross-validation and GA.

### 3.4. Model Parameter Setting

In this work, 240 groups of data are selected as training and testing samples for model development. The sum of the absolute values of the prediction errors of the training data is taken as the individual fitness value, and the smaller the individual fitness value, the better the individual is. 

**Figure 5:**
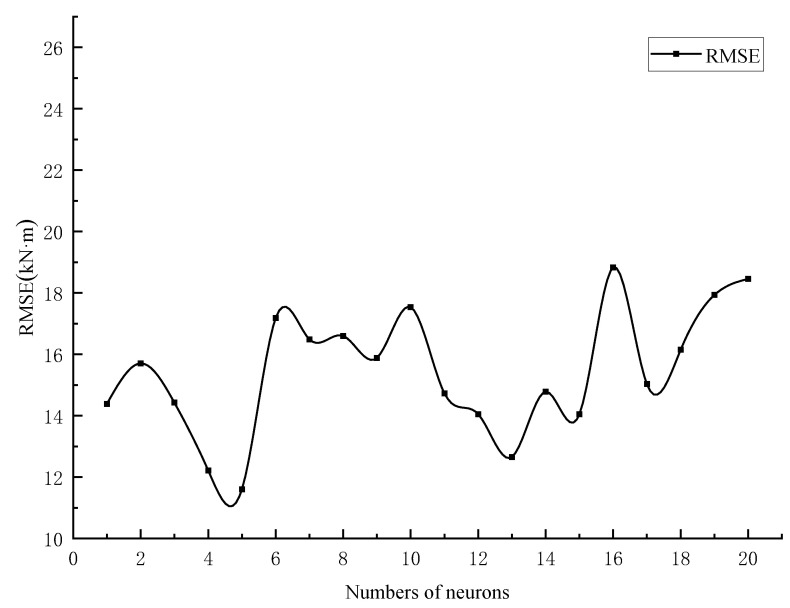
Selection for hidden layer neurons.

**Figure 6:**
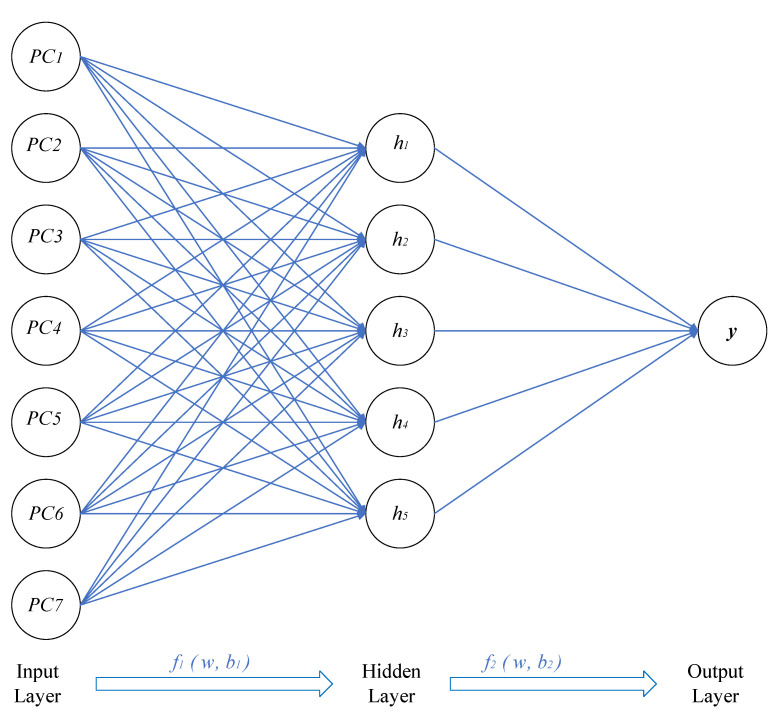
Back-propagation neural network (*PC*: principal component; *h*: hidden neuron; *y*: output).

To reach the optimal simulation of a BP neural network model, the number of hidden-layer neurons needs to be varied according to the learning rate, the number of neurons, the learning algorithm, etc., and to be determined after several experimental trials [26]. Additionally, according to the models and experimental methods from the literature [16,20,26], the number of neurons in the hidden layer was assumed to be in order from 1 to 20. In addition, the simulation results of BP neural network (BPNN) were used to test the optimal number of neurons (the prediction results are shown in Figure 5). In this study, the main objective is to improve the prediction model by the k-fold validation method. In this process, it is difficult to determine whether the prediction results have been changed by the k-fold validation method when the number of neurons in the hidden layer changes. Therefore, controlling the number of neurons in the hidden layer provides a more intuitive view of this approach. Figure 6 shows the final network architecture of ANN used in this study for torsional strength prediction.

In the BP neural network, the number of samples is randomly divided into two groups: The first group contains 170 samples for training and the remaining 70 samples were used as the testing samples. This is more indicative of the realism of the simulation results. In the GA-BP neural network, the number of samples is also divided, but the weights and thresholds are varied with the best gene individuals selected. The GA parameters are set as follows: The population size of GA is 10, the maximum iteration number is 50, the crossover rate is 0.4 and the mutation probability is 0.2.

### 3.5. Evaluation Metrics

In this paper, five statistical evaluation metrics were used to assess the performance of different models, which includes the mean absolute error (MAE), mean squared error (MSE), root mean squared error (RMSE), coefficient of determination ($R^{2}$) and mean absolute percentage error (MAPE) [38,39]. Those metrics are calculated as follows:

(6) $$\mathrm{MAE}=\frac{1}{n}\sum_{i=1}^{n}\left|{\hat{y}}_{i}-y_{i}\right|,$$

(7) $$\mathrm{MSE}=\frac{1}{n}\sum_{i=1}^{n}{\left({\hat{y}}_{i}-y_{i}\right)}^{2},$$

(8) $$\mathrm{RMSE}=\sqrt{\frac{1}{n}\sum_{i=1}^{n}{\left({\hat{y}}_{i}-y_{i}\right)}^{2}},$$

(9) $$R^{2}=\frac{\sum_{i=1}^{n}{\left({\hat{y}}_{i}-\bar{y}\right)}^{2}}{\sum_{i=1}^{n}{\left(y_{i}-\bar{y}\right)}^{2}},$$

(10) $$\mathrm{MAPE}=\frac{100\%}{n}\sum_{i=1}^{n}\left|\frac{{\hat{y}}_{i}-y_{i}}{y_{i}}\right|,$$

where n is the number of data groups, $\bar{y}$ is the mean of the testing torsional strength, ${\hat{y}}_{i}$ is the prediction of the torsional strength and $y_{i}$ is the testing torsional strength.

MSE, MAE and RMSE are convenient measures of the ‘mean error’ and are used to evaluate the degree of variability of the data. In addition to this, although RMSE is more complex and biased towards higher errors, it has a smoothed loss function. Furthermore, $R^{2}$ is used to characterize a good or bad fit by the variation in the data. Its normal range of values is [0 1], and the closer it is to 1, the better the variables of the equation explain y and the better the model fits the data. In addition, MAPE can also be used to determine how well different models evaluate the same data, with a value of 1 indicating a close relationship and 0 indicating a random relationship. The lower the value, the better the prediction.

## 4. Results and Discussion

The K-fold cross-validation method is used to sequentially select the training samples as the input data, and then the BP and GA-BP neural networks are used to predict the torsional strength of the RC beams. The results are shown in Table 4. From the table below, the results of the K-fold cross-validation for different 10 datasets are provided. In this step, the model with the best prediction performance is selected by comparing the evaluation metrics. Although some of the test groups have high correlation values closer to 1, they perform poorly in both the RMSE and MSE metrics and the values perform worse.

**Figure 7:**
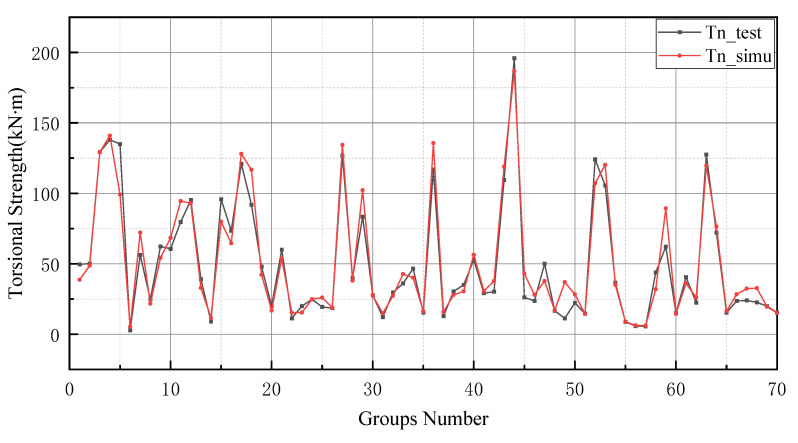
BP neural network optimized by k-fold cross-validation.

After the K-fold cross-validation is conducted, the results in Table 4 show that the best model should be group 10, as the MSE, RMSE and MAPE values of group 10 are lower than that of other models. In Table 4, the evaluation indicators can be used to assess the prediction performance of each group of models. The values of the MAE, RMSE, MSE and MAPE are smaller, and the generalization capacity of the prediction model is increased. Similarly, $R^{2}$ is also informative, and the value of the perfect model should be closer to 1. Therefore groups 2, 3, 4 and 5, where the MAE exceeds 10 kN ·m, should be excluded. Similarly, since the value of MSE for group 7 (542.109 ${\mathrm{kN}}^{2}\cdot m^{2}$) and group 8 (289.448 ${\mathrm{kN}}^{2}\cdot m^{2}$) is much greater than groups 1, 6, 9 and 10, the models in groups 7 and 8 should be excluded. In addition to this, the RMSE evaluation indicator provides a reason for excluding group 1, since the corresponding RMSE value for group 1 (11.241 kN·m) is larger than that for group 6 (9.083 kN·m), group 9 (7.977 kN·m) and group 10 (7.145 kN·m). The coefficient of 45.789% for group 9 in MAPE was much greater than that of group 6 (27.160%) and group 10 (18.477%). Finally, the coefficient of determination for group 10 (0.979) was closer to 1 than group 6 (0.968). Therefore, the tenth group is selected for comprehensive consideration. Furthermore, the weights and thresholds are recorded and inputted into the BP neural network model for comparison with the test set. After changing the initial weights and thresholds, the model prediction of the GA-BP neural network and the BP neural network is improved. However, this is not a significant improvement for the GA-BP neural network (Figure 7). 

**Figure 8:**
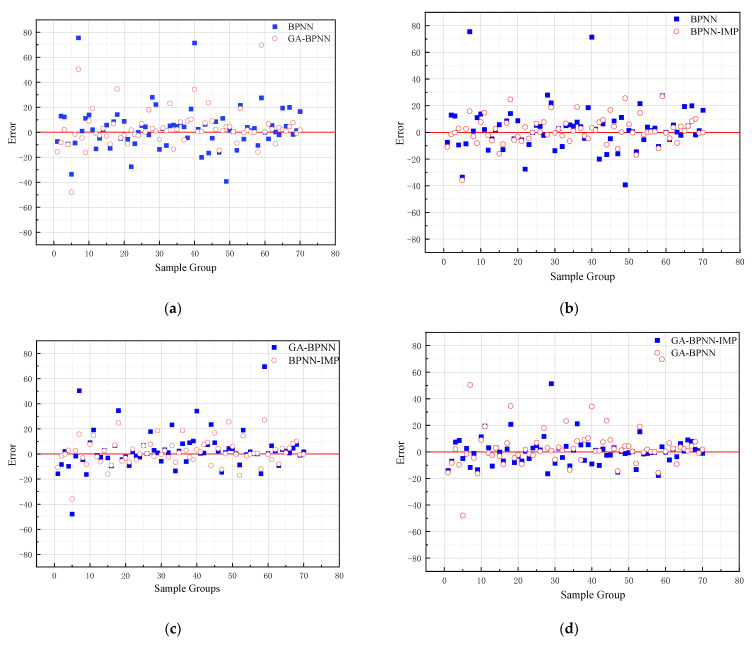

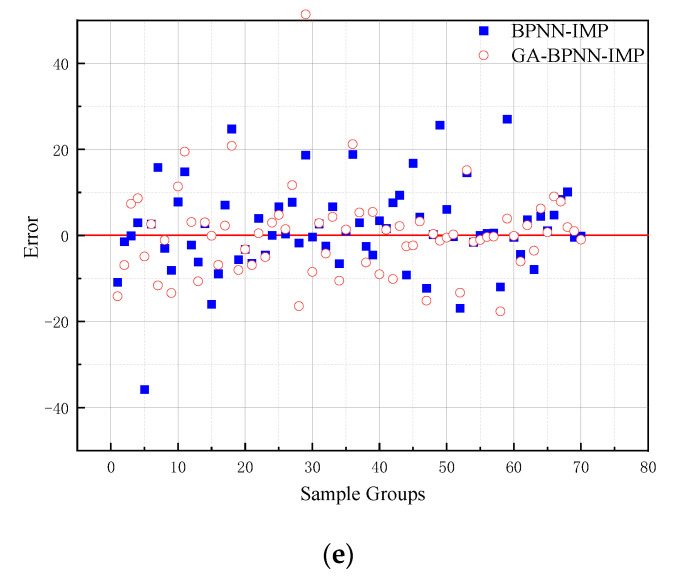
Error distribution between different models. (**a**) Error comparison between BPNN and GA-BPNN. (**b**) Error comparison between BPNN and optimized BPNN. (**c**) Error comparison between optimized BPNN and GA-BPNN. (**d**) Error comparison between GA-BPNN and optimized GA-BPNN. (**e**) Error comparison between optimized BPNN and optimized GA-BPNN.

From the figure below, GA and k-fold cross-validation perform well in improving the prediction accuracy of BPNN. The error range of the different models can be observed in Figure 8. In Figure 8a, the error of the GA-BPNN model is reduced from 78 kN·m to 50 kN·m. Similarly, Figure 8b shows the reduction in the model error from 78 kN·m to 40 kN·m using the k-fold validation method. In addition to this, the error was also reduced after using the k-fold validation method (Figure 8d). Furthermore, it can be observed from Figure 8c that the k-fold validation method outperforms the gene algorithm in terms of error reduction with 70 testing data. In addition, k-fold cross-validation has been reduced by approximately 15 kN·m in the absolute maximum error. Based on the K-fold cross-validation method, the prediction error values of BPNN and GA-BPNN are almost the same. Additionally, the maximum errors of both networks are close to approximately 25 kN·m.

**Figure 9:**
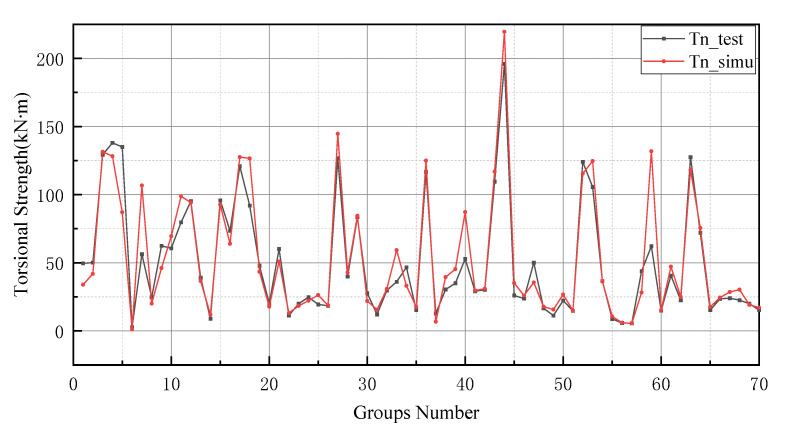
Prediction results of GA-BP neural network.

**Figure 10:**
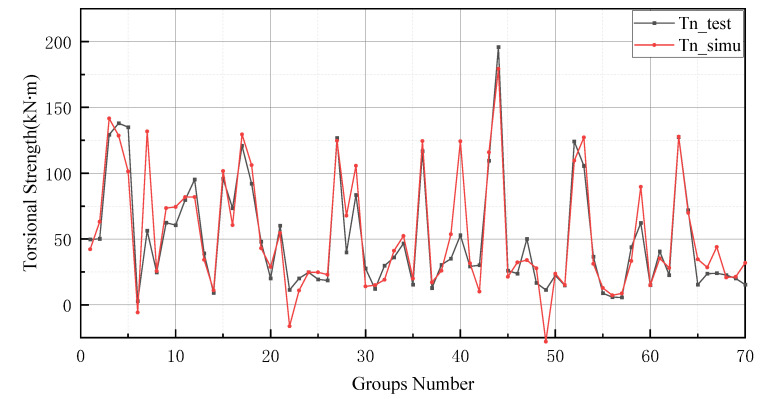
Prediction results of BP neural network.

The prediction values of the BP neural network model have higher error values than the -BP neural network. In particular, the BP neural network predicts negative values in the range of numbers 20 to 25 and numbers 45 to 50. In addition, the prediction of the GA-BP model and BP model has a low deviation range between 0 and 10. The reason for this result is the small number of data selected for training and the limited derivation of thresholds and weights by the BP neural network. It is worth noting that the BP neural network also performs well after optimizing the thresholds and weights by GA (as shown in Figure 9). Additionally, these two models produce better results in the last 10 testing data. In Figure 10, the simulation line shows high repeatability between the forecasted and actual values, especially in the range of group numbers 12–17, 40–45, 51–58 and 60–65. However, the optimized BP neural network model does not provide accurate prediction results of the groups of data for numbers 45–50. Comprehensively, the optimized BP neural network predictions show a high degree of agreement with the actual values. 

**Figure 11:**
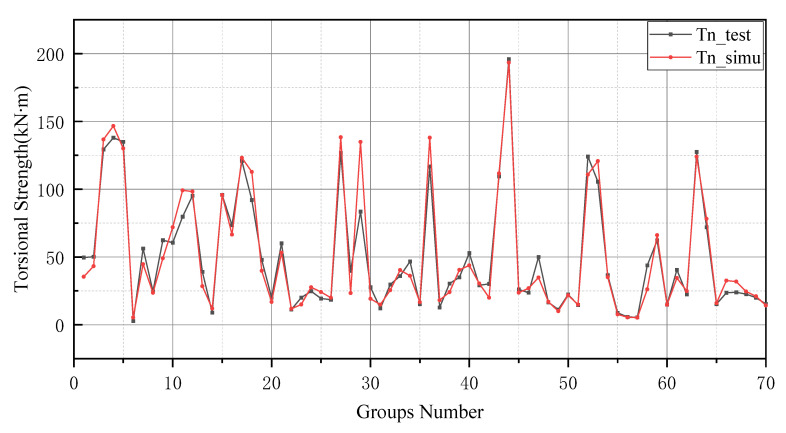
GA-BP neural network optimized by k-fold cross-validation.

**Figure 12:**
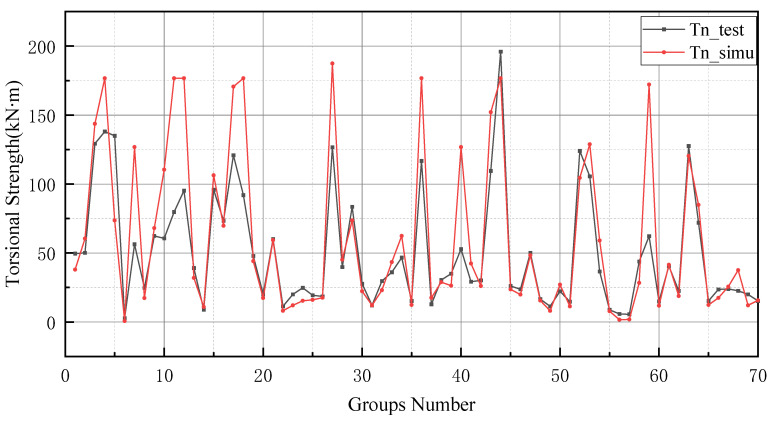
Prediction of torsional strength of ACI-318-14.

**Figure 13:**
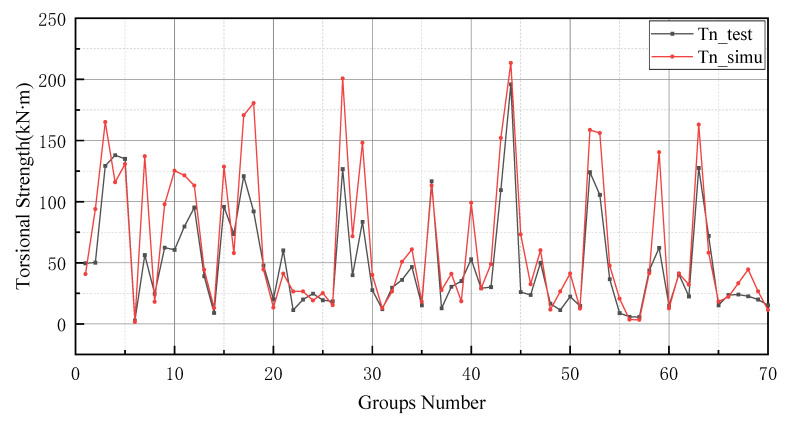
Prediction of torsional strength of BS-8110.

**Figure 14:**
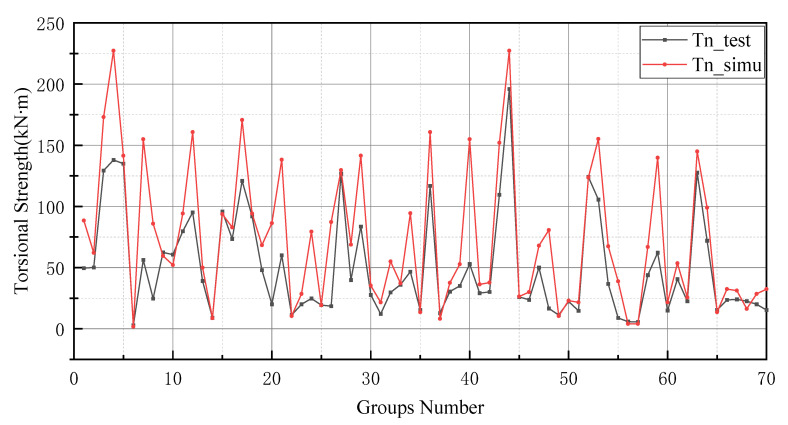
Prediction of torsional strength of TBC-500-2000.

**Figure 15:**
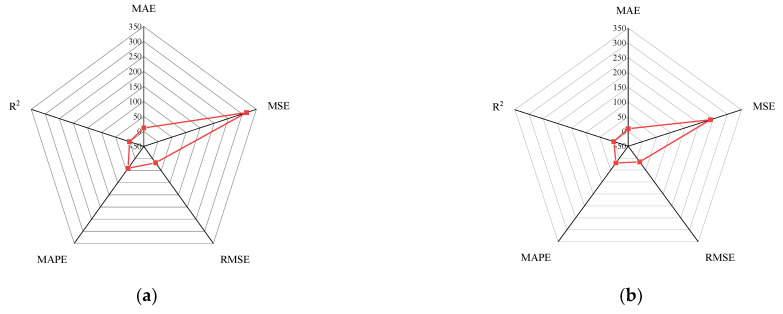

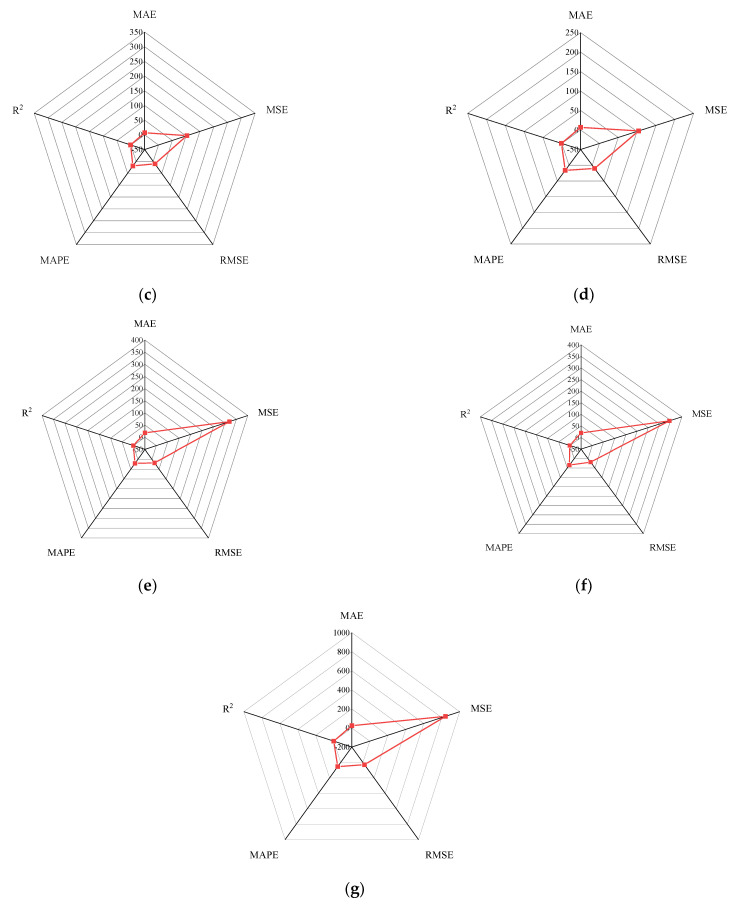
Radar diagram of calculation results. (**a**) BP neural network; (**b**) GA-BP neural network; (**c**) Optimized BP neural network; (**d**) Optimized GA-BP neural network; (**e**) ACI-318-14; (**f**) BS-8110; (**g**) TBC-500-2000.

In general, BP neural networks perform poorly without parameter optimization due to the random generation of thresholds and weights. Similarly, while GA can be employed to find the best weights and thresholds, the prediction results are often similar to BP neural networks when initializing the population. However, the network can be improved by inputting weights and thresholds that were filtered by k-fold cross-validation. This phenomenon has been validated via the samples (index 0 to 10) in Figure 8, Figure 9, Figure 10 and Figure 11. In Figure 8 and Figure 9, the prediction results of testing data of indices 1-6 are lower than their actual testing data, which is the opposite to the simulation results for the design code. The design code predictions are mostly higher than the actual values in this dataset, especially in TBC-500-2000 where the predicted lines almost include the actual lines. This phenomenon can also be seen in Figure 12, Figure 13 and Figure 14. Compared with the prediction of three building standards, the correlation of the coefficient of ACI-318-14 and BS-8110 is similar and it is higher than TBC-500-2000. The results in Table 5 show that the results from ACI-318-14 and BS-8110 are similar to the predictions of the BP neural network. This is due to the small sample datasets. In this case, most machine learning (e.g., decision tree, random forest, support vector machine linear, etc.) simulations are comparable to those of BP neural networks [11]. Figure 15 shows the radar diagram of evaluation metrics for model performance evaluation. However, the accuracy of the model is improved via GA optimization, with the MSE reduced from 315.363 to 240.046, but the $R^{2}$ only improved by about 0.04. In addition, the BP neural network optimized by k-fold cross-validation achieves better results than the BP neural network model. Additionally, the values of MAE, MSE, RMSE and MAPE are all reduced, and the value of the correlation coefficient increases by 0.1. This result is similar to that of the GA-BP neural network optimized by the k-fold cross-validation. Compared with the optimized GA-BP neural network, the k-fold cross-validation made a significant impact on the optimization of the GA-BP neural network by setting better initial weights and thresholds. The simulation results show that although GA also has an optimizing effect on the BP neural network, the improvement is neither adequate nor stable. However, the RMSE, MSE, MAE and MAPE values of the neural network model are reduced after optimization using the k-fold cross-validation method (Table 5). In particular, BPNN and GA-BPNN in the MSE evaluation metric decrease from 315.363 and 240.046 to 103.100 and 103.988, respectively, after optimization. The correlation coefficients of the BP neural network and GA-BP neural network models were also improved.

## 5. Conclusions

This study aims to investigate the performance of an optimized BP neural network in predicting the torsional strength of RC beams. Ten variables and four aspects were investigated in terms of section details, concrete strength, longitudinal bar and transvers bars. In this paper, to ensure the dataset is easier to use and to remove noise, the raw data are normalized using PCA and the seven most important features are retained for the prediction. The 240 groups of experimental data collected from existing publications were randomly divided into two groups: The first group contains 170 data samples for the model training and validation, and the remaining data were used to verify the accuracy of the model. the BP neural network was used in this paper, and the network parameters of this model were optimized using GA and k-fold cross-validation, respectively.

The design code is widely used in the construction sector as a traditional method for calculating the torsional strength of reinforced concrete beams. However, in order to obtain accurate predictions, the individual variables of a reinforced concrete beam are necessary, and the conditions of use of the beam under different conditions need to be taken into account. BPNN is able to ignore the conditions of application of the various variables for reinforced concrete beams and obtain predictions similar to those of the conventional design codes. This gives BPNN an advantage in the prediction of torsional forces in reinforced concrete beams. However, the method has limitations in terms of weights and thresholds. Due to the complexity of the construction conditions in reinforced concrete beams, it is difficult to obtain accurate and sufficient data. The application of k-fold cross-validation and GA methods can effectively avoid this situation. The k-fold cross-validation optimizes the initial threshold and weights of the BPNN after modelling 10 sets of data in turn. On the other hand, GA finds the optimal thresholds and weights in continuous iterations. While both methods improve the accuracy of the prediction results of the BPNN, k-fold cross-validation is more suitable for the case of insufficient data ($R^{2}$ increased from 0.846 to 0.943). At the same time, the GA-BPNN model is optimized on the basis of the thresholds and weights provided by k-fold cross-validation, and the improvement is significant. Based on the statistical results of MAE, MSE, RMSE, MAPE and $R^{2}$, the k-fold cross-validation-optimized GA-BPNN is the best prediction model for the torsional strength of reinforced concrete beams. 

In the future work, in addition to k-fold cross-validation-optimized GA-BPNN and existing design codes, other soft computing approaches, such as the support vector machine (SVM), extreme learning machine (ELM), adaptive neuro-fuzzy inference system (ANFIS), gene programming (GP), etc., will be investigated to compare and determine the optimal data-driven model for the torsional strength prediction of RC beam.

## Figures and Tables

**Table 1:** The range of input and output parameters (σ: Standard deviation).

Parameters	Input/Output	Unit	Minimum	Maximum	Average	σ
Section details	*b*	mm	85	600	265.943	124.295
	*h*	mm	178	600	391.155	134.699
	*b*′	mm	56.5	546	219.021	112.81
	*h*′	mm	149.5	549	336.241	123.514
Concrete	$f_{c}$	MPa	14.3	109.8	45.309	20.175
Longitudinal bar	$f_{yl}$	MPa	310	724	437.871	121.795
	$\rho_{l}$	Percentage	0.18	3.89	1.370	0.980
Transvers bar	$f_{yt}$	MPa	265	715	430.422	130.735
	$\rho_{t}$	Percentage	0.13	3.2	1.034	0.539
	*s*	mm	41	300	104.095	39.595
Test strength	$T_{u}$	kN·m	2.18	239	265.943	124.295

**Table 2:** Results of principal components analysis.

Parameters	PC1	PC2	PC3	PC4	PC5	PC6	PC7
b	0.4513	−0.0733	−0.1332	−0.0737	−0.2801	0.1528	0.3637
h	0.4105	−0.1879	−0.1967	0.2998	0.2935	−0.0503	−0.1717
$b^{\prime }$	0.4447	−0.0665	−0.14140	−0.1248	−0.2906	0.2082	0.3884
$h^{\prime }$	0.4029	−0.1520	−0.2235	0.3162	0.3667	−0.0109	−0.2833
$f_{c}{}^{\prime }$	0.1861	0.4078	0.14240	−0.4710	0.6869	0.1614	0.2243
$\rho_{l}$	−0.1359	0.4595	−0.0914	0.6491	0.1079	−0.1887	0.5298
$f_{yl}$	0.2955	0.4469	0.2218	0.0426	−0.2801	−0.1148	−0.1564
$\rho_{t}$	−0.2355	0.2810	−0.5069	0.0827	−0.0536	0.7372	−0.1903
$f_{yt}$	0.2680	0.4601	0.2442	0.0754	−0.2099	0.0434	−0.4526
s	0.0066	−0.2531	0.6923	0.3522	0.0862	0.5572	0.1033

**Table 3:** Building standards expression of torsional strength.

Building Standard	Expression for Torsional Strength	Reference
ACI-318-14	$T_{n}=\frac{2A_{o}A_{t}f_{yt}}{s}cot\theta$	$A_{t}$—the area of one leg of a closed stirrup resisting torsion. $A_{o}$—the gross area enclosed by shear flow path $f_{yt}$—characteristic strength of the links $A_{e}$—the cross-sectional area of the surrounding stirrups θ—the torsional angel
BS-8110	$T_{n}=\frac{0.8b^{\prime }h^{\prime }\left(0.87f_{yt}\right)A_{t}}{s}$	
TBC-500-2000	$T_{n}=\frac{2A_{o}A_{e}f_{yt}}{2\left(b^{\prime }+h^{\prime }\right)}$	

**Table 4:** Results of 10-fold cross-validation in BP neural networks.

Evaluation Metric	1	2	3	4	5	6	7	8	9	10
MAE (kN·m)	8.430	14.536	10.930	16.924	14.549	7.059	4.511	6.072	5.727	4.737
MSE $\left({\mathrm{kN}}^{2}\cdot m^{2}\right)$	126.362	333.467	230.512	1144.994	625.964	82.497	542.109	289.448	63.630	51.052
RMSE (kN·m)	11.241	18.261	15.183	33.838	25.019	9.083	23.283	17.013	7.977	7.145
MAPE (%)	33.530	33.431	38.752	30.469	19.198	27.160	16.307	17.697	45.789	18.477
$R^{2}$	0.945	0.896	0.756	0.840	0.776	0.968	0.979	0.952	0.952	0.979

**Table 5:** Calculation results of different models.

Models	MAE (kN·m)	MSE $\left(kN^{2}\cdot m^{2}\right)$	RMSE (kN·m)	MAPE (%)	$R^{2}$
BP neural networks	11.548	315.363	17.758	40.117	0.846
GA-BP neural networks	9.109	240.046	15.493	19.798	0.887
Optimized BP neural networks	7.063	103.100	10.154	18.957	0.943
Optimized GA-BP neural networks	6.742	103.988	10.197	16.251	0.950
ACI-318-14	17.832	320.016	17.889	20.205	0.867
BS-8110	19.700	344.436	18.559	34.507	0.856
TBC-500-2000	24.154	842.799	29.031	54.252	0.756

## Data Availability

The data presented in this study are available on request from the corresponding author.
